# Three-Dimensional Cobalt Hydroxide Hollow Cube/Vertical Nanosheets with High Desalination Capacity and Long-Term Performance Stability in Capacitive Deionization

**DOI:** 10.34133/2021/9754145

**Published:** 2021-10-26

**Authors:** Yuecheng Xiong, Fei Yu, Stefanie Arnold, Lei Wang, Volker Presser, Yifan Ren, Jie Ma

**Affiliations:** ^1^State Key Laboratory of Pollution Control and Resource Reuse, College of Environmental Science and Engineering, Tongji University, Shanghai 200092, China; ^2^Research Center for Environmental Functional Materials, College of Environmental Science and Engineering, Tongji University, 1239 Siping Road, Shanghai 200092, China; ^3^College of Marine Ecology and Environment, Shanghai Ocean University, Shanghai 201306, China; ^4^INM-Leibniz Institute for New Materials, 66123 Saarbrücken, Germany; ^5^Department of Materials Science and Engineering, Saarland University, 66123 Saarbrücken, Germany; ^6^Saarene-Saarland Center for Energy Materials and Sustainability, 66123 Saarbrücken, Germany; ^7^Shanghai Institute of Pollution Control and Ecological Security, Shanghai 200092, China

## Abstract

Faradaic electrode materials have significantly improved the performance of membrane capacitive deionization, which offers an opportunity to produce freshwater from seawater or brackish water in an energy-efficient way. However, Faradaic materials hold the drawbacks of slow desalination rate due to the intrinsic low ion diffusion kinetics and inferior stability arising from the volume expansion during ion intercalation, impeding the engineering application of capacitive deionization. Herein, a pseudocapacitive material with hollow architecture was prepared *via* template-etching method, namely, cuboid cobalt hydroxide, with fast desalination rate (3.3 mg (NaCl)·g^−1^ (h-Co(OH)_2_)·min^−1^ at 100 mA·g^−1^) and outstanding stability (90% capacity retention after 100 cycles). The hollow structure enables swift ion transport inside the material and keeps the electrode intact by alleviating the stress induced from volume expansion during the ion capture process, which is corroborated well by in situ electrochemical dilatometry and finite element simulation. Additionally, benefiting from the elimination of unreacted bulk material and vertical cobalt hydroxide nanosheets on the exterior surface, the synthesized material provides a high desalination capacity (117 ± 6 mg (NaCl)·g^−1^ (h-Co(OH)_2_) at 30 mA·g^−1^). This work provides a new strategy, constructing microscale hollow faradic configuration, to further boost the desalination performance of Faradaic materials.

## 1. Introduction

The recent two decades have witnessed soaring population growth, freshwater consumption, and global climate change, and ubiquitous pollution exacerbates freshwater deficiency [1, 2]. Therefore, scientists seek opportunities to produce usable water from inexhaustible seawater through desalination. Traditional desalination technologies, such as thermal distillation, reverse osmosis, and electrodialysis, exert heat, pressure, or electricity to extract water, which is highly energy-intensive [3]. Newly emerging processes, including capacitive deionization and membrane solar distillation, create less carbon footprint [4]. Capacitive deionization separates charged ions from saline media *via* electrosorption; the low applied cell voltage and high charge efficiency render this technique very promising regarding desalination of brackish water [5]. Distillation induced by concentrated solar radiation is intriguing because no external energy input is needed, but the freshwater production rate cannot meet large-scale production demand [6]. Therefore, capacitive deionization is attractive as an alternative energy-efficient desalination technique.

Electrodes are the core component of electrochemical desalination and ion separation. Typical electrodes can be divided into carbon electrodes and charge-transfer electrodes depending on the absence or presence of redox processes [7]. Ion intercalation or conversion reactions (charge-transfer materials) enable a much higher desalination capacity than possible for carbon-based electrodes because of the higher charge storage capacity and perm-selectivity; thereby, they enable facile desalination even at high molar strength. Currently, various charge-transfer materials for electrochemical desalination have been reported, such as transition metal oxides (MnO_2_, TiO_2_, V_2_O_5_, and ZnFe_2_O_4_) [8–11], Prussian blue analogs (nickel hexacyanoferrate) [12–14], polyanionic phosphates (Na_3_V_2_(PO_4_)_3_, Na_3_Ti_2_(PO_4_)_3_) [15–17], and two-dimensional materials (MXenes, MoS_2_, and TiS_2_) [18–22]. Among these materials, metal oxides or hydroxides hold the advantages of easy preparation, facile morphological manipulation, element diversity, and promising desalination performance. Yet, charge-transfer materials usually show a tradeoff between high capacity and good stability [23]. Theoretically, higher capacity means that more ions are removed, which generally causes more obvious volume changes. The performance degrades irreversibly if the material cannot relieve the accompanying stress [24]. For example, the lithiation of transition metal oxides (such as Fe_2_O_3_, Co_3_O_4_, and NiO) induces near 100% volume expansion in lithium-ion batteries, severely deteriorating the energy storage performance [25].

Stability is a vital characteristic of desalination electrodes. High stability means longer service life and more desalination cycles, which can decrease the cost of material and operation; meanwhile, less need for material signifies less solid waste produced, avoiding extra posttreatment costs. The chemical stability ensures that no toxic heavy metal ions (Mn^2+^, V^5+^, Co^2+^, etc.) from electrode materials leach into treated water, which is key to safe water quality. Therefore, electrode materials with good stability and reliability are crucial for desalination application.

The critical importance of performance stability can be addressed by designing flexible three-dimensional scaffolds to support Faradaic materials. For example, interconnected carbon networks and polymer hydrogel serve as structural support and pathways for electron transport [26]. Na_3_V_2_(PO_4_)_3_/graphene hybrid aerogel exhibits a high capacity of about 100 mg·g^−1^ after 50 cycles in a dual-ion deionization system but inferior stability than pure aerogel [26]. ZIF-67/PPy hybrid materials display stable desalination performance with a capacity of 11 mg·g^−1^ for 200 h [27]. However, the conductive network's cushion effect cannot enhance the intrinsic stability of charge-transfer materials, and simultaneous high capacity and good stability cannot be realized because supporting materials usually have lower desalination performance. Therefore, regulating the microscale or nanoscale structure of the Faradaic electrode materials will offer opportunities to further enhance the deionization performance instead of focusing on carbon-faradaic composite materials.

Constructing a hollow structure with lightweight architecture for pseudocapacitive materials is an appealing tactic to simultaneously achieve superior capacity and stability. A hollow configuration provides ample space for volume change, which has been widely applied in catalysis to strengthen the efficiency and cycling performance [28]. A hollow structure reduces the proportion of dead mass, which is the unreacted bulk of the material, thus improving the desalination capacity. Besides, hollow structures can ensure sufficient contact between electrode and electrolyte due to both inner and outer surfaces, providing more available active sites and enhancing the ion transport kinetics. An open graphene structure has been proposed for electrochemical desalination recently, but the inherent weakness of carbon materials restricts the performance [29]. Hollow charge-transfer electrode materials can break the tradeoff between high capacity and stable cycling, which is an appealing strategy to overcome shortcomings in capacitive deionization (low desalination capacity, low desalination rate, and poor stability) at a time.

Here, we synthesized a hollow transition hydroxide, cuboid cobalt hydroxide, with well-arranged nanosheets on its surface *via* template etching. Co(OH)_2_ exhibits substantial pseudocapacitance, widely utilized in the energy storage field [30, 31]. Amorphous Co(OH)_2_ nanocages or nanoboxes have also been used in batteries and catalytic water splitting, which employ similar electrochemical processes as electrochemical desalination [32, 33]. In our work, we show that cobalt hydroxide with optimized morphology provides a high desalination capacity of 117 ± 6 mg (NaCl)·g^−1^ (h-Co(OH)_2_) and a fast desalination rate of 3.3 mg (NaCl)·g^−1^ (h-Co(OH)_2_)·min^−1^. The cobalt hydroxide electrodes exhibit excellent stability without the coupling of three-dimensional supporting networks. This paper offers a new strategy to elevate the desalination rate and strengthen the stability through the hollow structure without sacrificing the high capacity of faradic electrodes, showing the promising application in large-scale CDI.

## 2. Materials and Methods

### 2.1. Preparation of the Cu_2_O Template

All chemicals and reagents were purchased from Sinopharm Chemical Reagent Co., Ltd, and used without further purification. CuCl_2_·2H_2_O (10 mM, 100 mL) was heated in a 55°C water bath under magnetic stirring, and then, NaOH (2 M, 10 mL) was added slowly. The color of the solution changed from dusty aquamarine to dark brown. 30 min later, freshly prepared ascorbic acid (0.6 M, 10 mL) was added dropwise by a syringe, and the solution appeared brownish-black, turning into brick red in the end. After continuous agitation for 3 h, the Cu_2_O template was separated by vacuum filtration and washed with deionized water and ethanol several times. The sample was acquired after 60°C of vacuum drying for 5 h.

### 2.2. Preparation of Hollow Co(OH)_2_ Cube/Vertical Nanosheets

100 mg Cu_2_O powder was added into a mixed solution of 100 mL deionized water plus 100 mL ethanol, and the mixture was ultrasonicated for 1 h. The remaining steps were conducted under 500 rpm magnetic stirring. 6.6 g polyvinylpyrrolidone K30 (PVP-K30) was added. After 30 min, 60 mg CoCl_2_·6H_2_O was added. Another 30 min later, Na_2_S_2_O_3_ (1 M, 100 mL) was added dropwise by a peristaltic pump with a controlled rate of one drop per second. When the solution turned into jade green, the hollow cube cobalt hydroxide material was obtained after vacuum filtration, cleaning, and drying at 40°C overnight. Regarding our manuscript, we refer to hollow Co(OH)_2_ cube as “h-Co(OH)_2_.”

### 2.3. Electrode Preparation

The active materials (Co(OH)_2_ powder or activated carbon) were mixed with a conductive additive (acetylene black) and binder (polyvinylidene difluoride, PVDF) at a mass ratio of 8 : 1 : 1. Afterward, 1-methyl-2-pyrrolidinone (NMP) was added appropriately, and the mixture was stirred overnight to form a homogeneous and viscous paste. Then, electrodes were prepared by the doctor-blade method on graphite paper with a thickness of ca. 75 *μ*m. Next, prepared electrodes were dried at 40°C under vacuum for 12 h to remove any residual organic solvent. The electrode's mass was determined by the difference between pure graphite paper and dried electrode, and the electrode mass used for electrochemical desalination was about 4.0 mg.

### 2.4. Electrochemical Characterization

All electrochemical characterization, including cyclic voltammetry (CV), galvanostatic charging/discharging cycling (GCD), and electrochemical impedance spectroscopy (EIS), was conducted by the electrochemical station (CHI660D, Shanghai Chenhua Instruments Co.) in a three-electrode cell with 1 M NaCl as the electrolyte. Electrodes of 1*x*1 cm^2^ were used as the working electrode, while Pt and Ag/AgCl were employed as the counter electrode and reference electrode, respectively. Cyclic voltammetry was swept between -0.6 V and +0.6 V under various scan rates (1-50 mV·s^−1^), and GCD was tested under the same voltage window with different specific currents (0.1-2 A·g^−1^). The EIS spectra were recorded over the frequency range of 10^5^ Hz to 10^−2^ Hz with an amplitude of 5 mV. Besides, CV and GCD were also carried out in a two-electrode cell with a working electrode and an oversized activated carbon powder (400 mesh, Macklin) electrode as the counter electrode in 1 M NaCl.

In situ electrochemical dilatometry was performed in an ECD-3 nanoelectrochemical dilatometer (EL-CELL) to track the volume changes during cyclic voltammetry by constructing a two-electrode cell (h-Co(OH)_2_ versus carbon) with an oversized activated carbon (90 mass% YP-80F, 10 mass% polymer binder) as counter- and quasireference electrode in 1 M Na_2_SO_4_. All cell parts were dried overnight at 80°C and introduced into an argon-filled glovebox (MBraun Labmaster 130; O_2_; H_2_O < 0.1 ppm). h-Co(OH)_2_ electrode was drop-casted on a Pt disc with a diameter of 10 mm, and the initial thickness was 65 *μ*m. All electrochemical measurements were carried out at a climate chamber (Binder) with a constant temperature of 25 ± 1°C. Cyclic voltammetry (CV) measurements were carried out using a VMP3 multichannel potentiostat/galvanostat from Bio-Logic (France). After a resting period and stabilization time of 48 h or 72 h, cyclic voltammograms were recorded at 1 mV s^−1^ in the range of -0.8 V to +0.3 V vs. carbon.

### 2.5. Electrochemical Desalination Performance

The flow-by capacitive deionization device consists of the following parts: acrylic plates, silicone gaskets, electrodes, cation/anion exchange membranes, and a chamber with the dimension of 2 × 2 × 1 cm^3^. The whole testing system is composed of a power source (LAND battery testing system), deionization apparatus, peristaltic pump, NaCl solution tank, and conductivity meter (Mettler Toledo S230). All deionization tests were done in constant current operation and batch mode. The NaCl solution volume was 25 mL, and the flow rate was fixed at 15 mL·min^−1^. Other parameters, including cutoff voltage (±0.6 V, ±0.8 V, ±1.0 V, ±1.2 V, ±1.4 V, and ±1.6 V), initial feed concentration (10 mM, 100 mM, and 600 mM), and specific current (30-100 A·g^−1^), were adjusted to acquire the optimized performance and investigate the desalination mechanism. The deionization capacity was calculated based on the relationship between conductivity and concentration and is presented in the following equation:

(1) $$\begin{array}{c} \text{Deionization capacity }Q=\frac{\left(C_{i}-C_{f}\right)V}{m}\;\left(\frac{{\text{mg}}_{\text{NaCl}}}{{\text{g}}_{\text{h}-\text{Co}{\left(\text{OH}\right)}_{2}}}\right), \end{array}$$

where *C*_*i*_ and *C*_*f*_ (mg·L^−1^) are the concentration of NaCl before and after deionization, respectively; *V* (25 mL) is the volume of NaCl solution; and *m* (mg) is the total mass of Co(OH)_2_ electrode containing conductive additive and binder.

### 2.6. Material Characterization

The formation process and surface morphology of hollow Co(OH)_2_ were characterized by scanning electron microscopy (SEM, Hitachi S-4800) and transmission electron microscopy (TEM, JEOL-2010F). The crystal structure was analyzed using X-ray diffraction (XRD D8 ADVANCE, Bruker AXS) operated at 40 mA and 45 kV with Cu-K*α* radiation (*λ* = 0.154 nm, 5°/min). X-ray photoelectron spectroscopy (XPS) analysis was carried out in a ThermoFisher ESCALAB 250Xi spectrometer using Al-K*α* radiation (1486.6 eV) with a base pressure of 1 × 10^9^ torr. The peak energies were calibrated by C1s peak at 284.8 eV. The wettability was reflected by the water contact angle (POWEREACH JC2000D2W). Raman spectra were recorded with a Renishaw inVia system using an Nd:YAG laser with an excitation wavelength of 532 nm. The spectral resolution was 1.2 cm^−1^, and the diameter of the laser spot on the sample was around 2 *μ*m with a total power exposure of 0.5 mW. The exposure time is 30 s with 5 accumulations. The numeric aperture was 0.75. The specific surface area and pore size distribution were calculated from the adsorption/desorption isotherms of N_2_ at -196°C by the multipoint BET and BJH method using a BELSORP Max instrument (BEL), and the sample was degassed at 120°C for 6 h before the measurements. The 2D-NLDFT analysis was carried out with SAIEUS assuming finite pores with an aspect ratio of 6 and using a regularization parameter of 2.75.

## 3. Results and Discussion

### 3.1. Material Characterization

**Figure 1:**
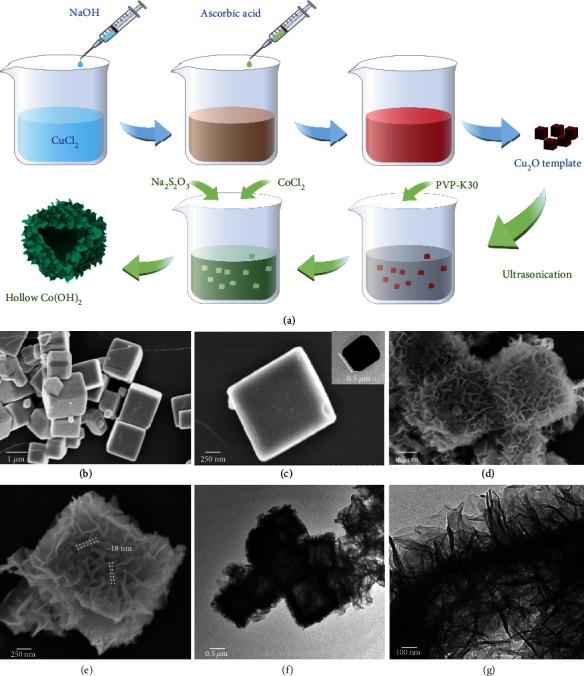
(a) Schematic illustration of the hollow Co(OH)_2_ cube preparation. Scanning electron micrographs of the Cu_2_O (b, c) (inset in (c) is the transmission electron micrograph of Cu_2_O cube) and hollow Co(OH)_2_ cube (d, e). Transmission electron micrographs of the hollow Co(OH)_2_ cube (f, g).

Hollow Co(OH)_2_ cube was synthesized *via* classical coordination etching and precipitation process guided under Pearson's hard and soft acid-base principle [34, 35], with the schematic illustration shown in Figure 1(a).

First, the Cu_2_O sacrificial template was prepared by precipitation and reduction reaction, exhibiting a cubic shape with a side length of 0.5 *μ*m to 1 *μ*m (Figure 1(b)), and the surface of the Cu_2_O cube is relatively smooth (Figure 1(c)). Afterward, Na_2_S_2_O_3_ was used to etch the precursor and release OH^−^ to create an alkaline environment, whereby cobalt hydroxide was precipitated synchronously. The simultaneous etching and Co(OH)_2_ growing process is verified by time-dependent TEM images (Supporting Information, Fig. S1). The appearance of cobalt hydroxide is cuboid with an apparent hollow structure (Figure 1(f)). Some individual cubes are broken, which exposes the interior surface and shortens the ion transport distance during electrochemical operation. Thin cobalt hydroxide nanosheet arrays with a thickness of around 18 nm extend from the shell, providing abundant sites for ions to be reacted and stored (Figures 1(d), 1(e), and 1(g)).

**Figure 2:**
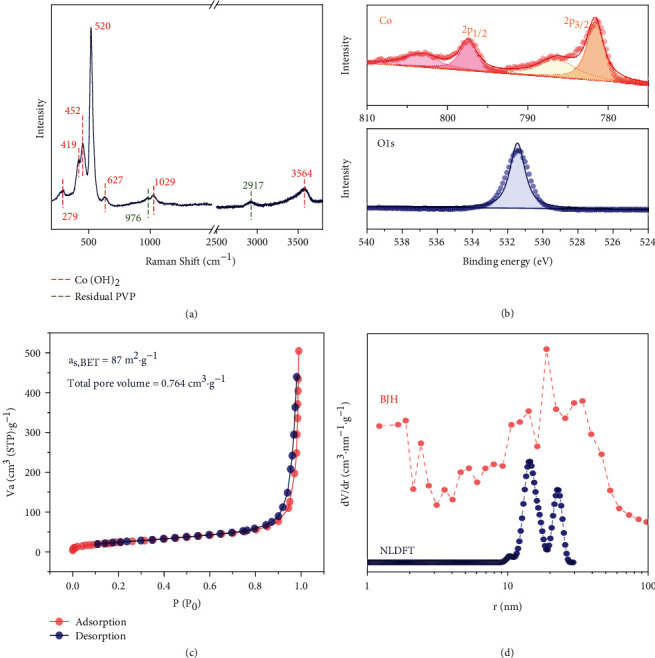
Characterization data of hollow Co(OH)_2_ cubes: Raman spectrum (a), X-ray photoelectron spectra of Co 2p and O 1 s (b), N_2_ adsorption/desorption isotherm measured at -196°C (c), and pore size distribution based on BJH and NLDFT models (d).

The successful synthesis of cobalt hydroxide in the form of Co(OH)_2_ was confirmed by Raman spectroscopy (Figure 2(a)). The peak at 279, 419, 452, 520, 1029, and 3564 cm^−1^ belongs to Co(OH)_2_ [36, 37]; among them, the band at 520 cm^−1^ attributes to the CoO(A_g_) symmetric stretching mode; the bands at 452 cm^−1^ and 3564 cm^−1^ are assigned to the OCoO bending mode [37] and OH vibrational mode [38], respectively. The peak at 627 cm^−1^ corresponds to the F^22^_g_ of Co_3_O_4_ or belongs to Co(OH)_2_ [39], while the bonds at 976 cm^−1^ and 2917 cm^−1^ possibly arise from the remaining PVP [40, 41]. Besides, none of the characteristic reflections for CoO are found after 40°C vacuum drying. Based on the XPS results (Figure 2(b)), the prominent peak of Co 2p_3/2_ locates at around 781.5 eV with a satellite peak ca. 5 eV away. This spectral data verifies the existence of Co^2+^, which is further corroborated by a single peak of O 1s spectra at approximately 531.4 eV. [42] Despite no subpeak for hydroxyls, the hydrophilicity of Co(OH)_2_ electrode is verified by decreasing water contact angle with time. After desalination, a contact angle of 54.40° was measured (Supporting Information, Fig. S2).

N_2_ adsorption/desorption isotherms show the type V curve, indicating no micropore but mesopore structure exists in h-Co(OH)_2_, and a type H3 hysteresis loop suggests slit pore geometry created by the stacking of flake particles (Figure 2(c)). The specific surface area of the Co(OH)_2_ cube determined by the Brunauer-Emmett-Teller (BET) method is about 87 m^2^·g^−1^, and the mean pore radius derived from the Barrett-Joyner-Halenda (BJH) and NLDFT (2D-NLDFT finite pores were assumed) models is about 20 nm. Such large mesopores are expected to yield sufficient space for ion transport pathways during electrochemical desalination (Figure 2(d)).

### 3.2. Electrochemical Performance

**Figure 3:**
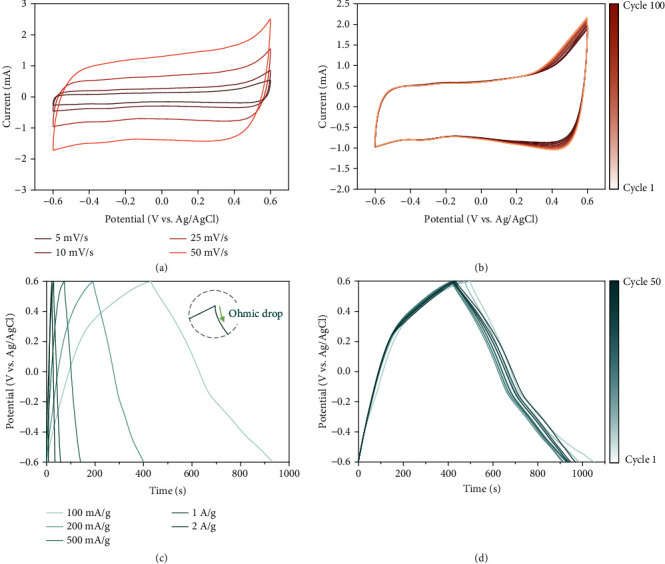
(a) Cyclic voltammograms of the hollow Co(OH)_2_ cube at different scan rates. (b) 100 cyclic voltammetry cycling curves at 25 mV·s^−1^. (c) Galvanostatic charging/discharging profiles of the hollow Co(OH)_2_ cube at different specific currents. (d) 50 galvanostatic charging/discharging cycling profiles at 100 mA·g^−1^.

The cyclic voltammogram of h-Co(OH)_2_ presents a quasirectangular shape without observable redox peaks (Figure 3(a)). This specific shape indicates that h-Co(OH)_2_ is a pseudocapacitive material like other transition metal oxides and hydroxides corroborated by GCD profiles without plateau (Figure 3(c)). A pair of shallow peaks at around -0.3 V and -0.1 V is evident at a rate of 1 mV·s^−1^ (Supporting Information, Fig. S3), which aligns with the pseudocapacitive property due to the small Δ*V* between redox peaks [43]. Besides, the thin-film electrode is prone to be pseudocapacitive as a result of free ion diffusion [44]. This aligns with the open (hollow) architecture and short diffusion paths within h-Co(OH)_2_. The capacitance values calculated from different scan rates (5-50 mV·s^−1^) are 32-24 F·g^−1^. The slight loss of capacitance at a high scan rate demonstrates the rapid charge storage mechanism.

The h-Co(OH)_2_ electrode shows very good performance stability, characterized by repeated cyclic voltammetry and galvanostatic charge/discharge cycling. The cyclic voltammograms' shapes display no significant change after 100 cycles except minor alteration at +0.4 V to +0.6 V (vs. Ag/AgCl). Hence, h-Co(OH)_2_ might experience side reactions at a voltage beyond +0.4 V (Figure 3(b)). The performance from GCD profiles degrades slightly at first and returns to the original level, and the retention proportion for charging capacity is 97% after 50 cycles (Figure 3(d)).

To better predict the performance during deionization, we carried out experiments in a two-electrode configuration (Supporting Information, Fig. S4). The cyclic voltammograms show some broad redox peaks as the indicator of the valence change between Co^2+^ and Co^3+^. The electrode polarization occurs when the cell voltage exceeds 0.8 V, which might be due to slight carbon oxidation and water hydrolysis. The charging capacity at different rates (40-1000 mA·g^−1^) changes moderately from 35 mAh·g^−1^ to 21 mAh·g^−1^. This behavior is like what was observed during cyclic voltammetry. The h-Co(OH)_2_ electrodes provide an excellent rate capability and stability, projecting to have relatively high deionization capacity and good durability, and the reaction equation of Na^+^ capture is stated as follows in Equations (2) and (3) [11, 31].

(2) $$\begin{array}{c} \text{CoOOH}+x{\text{e}}^{-}+x{\text{Na}}^{+}⟶\text{Co}{\left(\text{OH}\right)}_{2}{\text{Na}}_{x} \end{array}$$

(3) $$\begin{array}{c} \text{Co}{\left(\text{OH}\right)}_{2}{{\left(\text{OH}\right)}_{x}}^{-}+x{\text{Na}}^{+}⟶\text{Co}{\left(\text{OH}\right)}_{2}{\left(\text{OH}\right)}_{x}{\text{Na}}_{x} \end{array}$$

### 3.3. Desalination Performance

**Figure 4:**
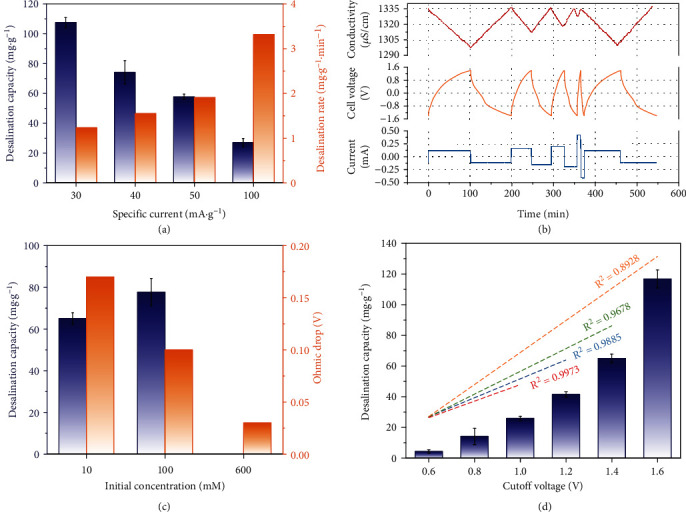
(a) Desalination capacity and desalination rate at different specific currents (initial NaCl concentration: 10 mM; cutoff voltage: ±1.4 V). (b) Recorded profiles of the conductivity, cell voltage, and current data at different rates (30/40/50/100/30 mA·g^−1^). (c) Desalination capacity and ohmic drop at different initial NaCl concentrations (specific current: 30 mA·g^−1^; cutoff voltage: ±1.4 V). (d) Desalination capacity with changing cutoff voltages and the correlation between capacity and cutoff voltage (initial concentration: 10 mM; specific current: 30 mA·g^−1^).

Hybrid capacitive deionization (HCDI) cell is applied to evaluate the desalination performance of h-Co(OH)_2_ under various conditions, and this classical configuration adopts ion exchange membranes to strengthen the charge efficiency and oxygen penetration. The specific current is a vital operational parameter, especially for engineering practices, closely related to desalination efficiency and energy consumption. The desalination capacity is 107 ± 3 mg (NaCl)·g^−1^ (h-Co(OH)_2_) at a rate of 30 mA·g^−1^, and the capacity declines as the specific current increases to 100 mA·g^−1^ (Figure 4(a)). Higher values of the specific current result in a shorter charging time and greater diffusion limit, and fewer active sites are available, leading to lower capacity (Figure 4(b)). As the specific current returns to the original level, the capacity is mostly restored [45], as shown in Supporting Information, Fig. S5. A higher specific current translates to a faster charge transfer, resulting in a higher desalination rate, and this value is 3.3 mg·g^−1^·min^−1^ at 100 mA·g^−1^, almost three times more than the rate at 30 mA·g^−1^ (Figure 4(a)). The energy consumption increases with a higher specific current, confirmed by lower charge efficiency, which drops to 93% at 100 mA·g^−1^ (Supporting Information, Fig. S6).

The NaCl concentration also affects the desalination capacity, increasing from 65 ± 3 mg·g^−1^ to 78 ± 7 mg·g^−1^ when the NaCl feedwater concentration increases from 10 mM to 100 mM (Figure 4(c) and Supporting Information, Fig. S7A). The positive effect of increasing NaCl concentration can be explained by declining ohmic drop, and this value derived from the voltage profile is 0.17 V, 0.1 V, and 0.03 V for 10 mM, 100 mM, and 600 mM NaCl, respectively (Figure 4(c)).

The desalination capacity increases from 4 ± 1 mg·g^−1^ to 65 ± 3 mg·g^−1^ when the voltage range expands from -0.6 V/+0.6 V to -1.4 V/+1.4 V (Figure 4(d) and Supporting Information, Fig. S7B). Broader voltage intervals correspond with prolonged charging times, as shown in Supporting Information, Fig. S8. Thus, more charge is accumulated at the electrode to participate in the desalination process. Some of the invested charge is consumed due to the internal cell resistance (intrinsic electrode resistance, electrode-solution contact resistance, and electrolyte resistance), and therefore, the capacity under -0.6 V/+0.6 V is small. When the voltage is further widened to -1.6 V/+1.6 V, the desalination capacity reaches 117 ± 6 mg·g^−1^. As shown in the CDI Ragone plot (known as Kim-Yoon plot; Supporting Information, Fig. S9), h-Co(OH)_2_ shows better desalination performance than commercial Co(OH)_2_ nanosheet, proving the positive effect of hollow configuration. Moreover, h-Co(OH)_2_ presents higher capacity than pure Co(OH)_2_ on graphite paper (48 mg·g^−1^) and interconnected hollow graphene shell (14 mg·g^−1^) proposed recently [29, 46].

A large effective surface area estimated to be around 179.8 m^2^·g^−1^, over the value from N_2_ adsorption, is required if all Na^+^ is adsorbed at the external surface of h-Co(OH)_2_ [47]. This rough deduction indicates that the subsurface also participates in deionization, underscoring the pseudocapacitive property. The relationship between average capacity and cutoff voltage is matched well by linear regression (Figure 4(d)), while the linear fit yields a larger deviation from the linear correlation at higher voltages as Faradaic side reactions intensify at 1.6 V [48].

**Figure 5:**
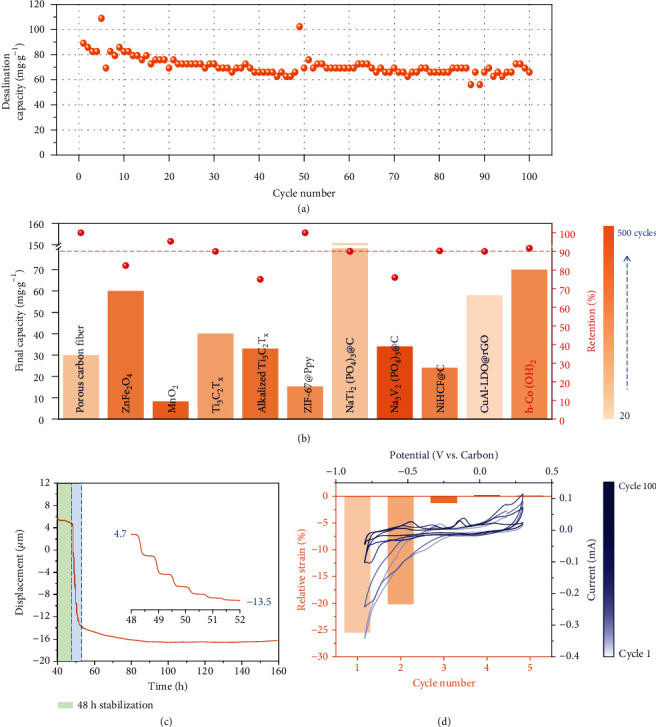
(a) Desalination capacity of HCDI cell for 100 cycles (specific current: 30 mA·g^−1^; cutoff voltage: ±1.2 V; initial NaCl concentration: 10 mM). (b) Stability comparison of various electrode materials considering final capacity, retention rate, and cycle number (a different color represents the cycle number) [11, 16, 17, 27, 47, 49–53]. (c) h-Co(OH)_2_ electrode displacement development during in situ electrochemical dilatometry in CV mode, and the inset is the magnified diagram for the initial 4 h. (d) Cyclic voltammograms and relative strain at the 1^st^, 3^rd^, 10^th^, 50^th^, and 100^th^ cycle.

Furthermore, 100 desalination cycles were carried out to characterize the performance stability of h-Co(OH)_2_ electrodes. The average desalination capacity is about 70 mg·g^−1^, with a retention of 92% (Figure 5(a)). Compared with other carbon-metal composite electrodes, h-Co(OH)_2_ exhibits a high capacity and good stability simultaneously (Figure 5(b)). Besides, the desalination rate of h-Co(OH)_2_ electrode is comparable to or even higher than advanced Faradic materials proposed recently (Supporting Information, Table S1). Subsequently, the electrochemical properties were also characterized after desalination. Cyclic voltammograms and galvanostatic charge/discharge profiles after desalination at different cutoff voltages and specific currents are analogous to raw electrodes except for minor peak alteration at around -0.3 V. Electrochemical impedance spectra exhibit similar internal ohmic resistance and charge-transfer resistance (Supporting Information, Fig. S10). The electrodes, after desalination at different rates, display slightly higher capacitance and smaller charge-transfer resistance. Therefore, h-Co(OH)_2_ electrodes demonstrate good electrochemical stability. Apart from Na^+^ removal, h-Co(OH)_2_ is possible to intercalate Cl^−^ by applying h-Co(OH)_2_ as an anode, supported by its higher chlorine ion removal capacity compared with activated carbon (Supporting Information, Fig. S11-S15).

The stability of h-Co(OH)_2_ electrodes is further illustrated by in situ electrochemical dilatometry, an indicator of volume change during sodiation/desodiation process and applied in two-dimensional Ti_3_C_2_-type MXene, and the schematic illustration of a dilatometer is presented in Supporting Information, Fig. S16 [27, 54, 55]. To match the voltage window vs. Ag/AgCl, the voltage range was set as -0.8 V to +0.3 V (vs. carbon), and 1 M Na_2_SO_4_ electrolyte was used to avoid chlorine-related corrosion of the apparatus. Before testing, the cell was stabilized for 48 h, and over 80% of the electrode displacement occurs at the initial 4 h (1^st^ to 7^th^ cycle), following the decay trend displayed in long-cycle desalination (Figure 5(c)). The drop in the displacement may align with the partial disintegration of hollow Co(OH)_2_ cubes. The displacement remains almost unchanged afterward, suggesting that the h-Co(OH)_2_ electrode has minimal volume change during salination/desalination. Figure 5(d) displays cyclic voltammograms, which were recorded during the in situ dilatometry. The shape of the cyclic voltammogram of the h-Co(OH)_2_ electrodes closely matched with what was recorded in a typical standard three-electrode test cell. All obtained electrochemical features are similar to those of the cyclic voltammetry results presented before, even though the current is comparably low in the dilatometry cell. Their shape remains stable after the third cycle. Correspondingly, the relative strain becomes negligible (the original data of relative strain is presented in Supporting Information, Fig. S17A), demonstrating that the hollow structure relieves the stress from volume change appropriately. To exclude the influence of the stabilization period, the system was tested after stabilizing for 72 h. The relative strain tends to be stable, and the relative strain becomes zero after the 10^th^ cycle (Supporting Information, Fig. S17B). Therefore, electrochemical dilatometry proves that the hollow h-Co(OH)_2_ structure can relieve the electrode volume change to enhance the stability after the desalination has reached equilibrium.

### 3.4. Desalination Mechanism

The intrinsic pseudocapacitance provides maximum synergy with the fast ion transport *via* the porous 3D architecture of the hollow Co(OH)_2_ cubes. These beneficial electrochemical properties result in a high desalination capacity and good stability. A kinetic analysis can further investigate the charge storage and ion removal kinetics. Specifically, one can derive key information via analysis of the dependency of the measured current with the sweep rate [56], stated in equations (4) and (5).

(4) $$\begin{array}{c} i={av}^{b}, \end{array}$$

(5) $$\begin{array}{c} \log\left(i\right)=b\log\left(v\right)+\log\left(a\right). \end{array}$$

**Figure 6:**
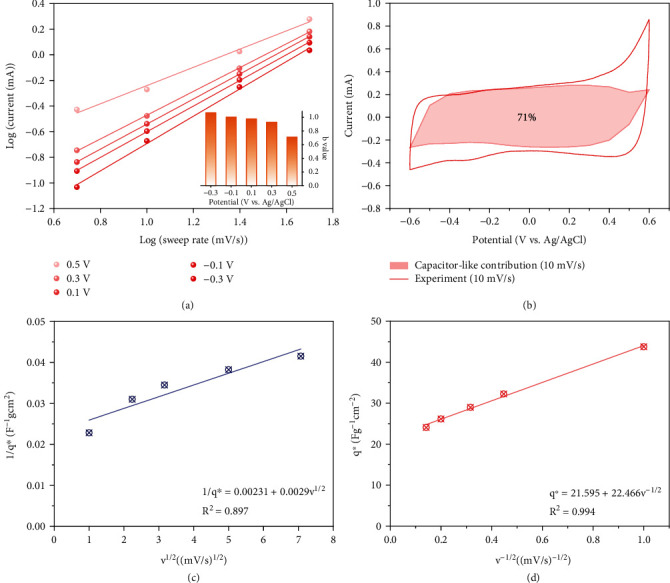
(a) Calculation of *b*-values based on CV curves. (b) Estimation of pseudocapacitive contribution of the cyclic voltammogram at 10 mV·s^−1^. (c) The relationship between 1/*q*^∗^ and *v*^1/2^. (d) The relationship between *q*^∗^ and *v*^−1/2^.

The *b*-value can be determined from the plot's slope between log(*i*) and log(*v*) and indicates the charge storage mechanism. When the *b*-value is 0.5, we have a system limited by diffusion, as typically found for battery-like systems via ion intercalation. In contrast, a *b*-value of 1.0 is found for an ideal capacitor, such as an electrical double-layer capacitor, where charge storage is enabled via ion electrosorption. According to Figure 6(a), the calculated *b*-values exceed 0.8 when the voltage is lower than 0.3 V. This very high value aligns with the pronounced pseudocapacitive behavior of the electrode material. The *b*-value drops to about 0.7 at 0.5 V, which agrees with the more battery-like process through Na^+^ intercalation and side reactions. This also aligns with the findings from the performance stability measurements via continued cyclic voltammetric testing.

It is also possible to quantify the percentage of surface-controlled capacitance (capacitor-like contribution) corresponding with either a perfect diffusion-limited system (*b*-value of 0.5) or a perfect capacitor (*b*-value of 1.0). The closer it is to one, the more perfect is the pseudocapacitive response. A beneficial calculation for this consideration is the use of Equation (6) which is often referred to as Dunn analysis [44]:

(6) $$\begin{array}{c} i\left(V\right)=k_{1}v+k_{2}v^{1/2}. \end{array}$$

In this equation, *k*_1_ corresponds with an ideal (pseudo)capacitive contribution and *k*_2_ with a battery-like feature. For our electrode material, *k*_1_ represents 71% of the total capacity at the scan rate of 10 mV·s^−1^ (Figure 6(b)). As a comparison, this percentage for a recently reported black phosphorus composite is 59% at 5 mV·s^−1^ [57].

An alternative analysis is the Trasatti method. This approach differentiates an “inner” and “outer” surface-controlled capacity of h-Co(OH)_2_ electrode [58]. To be specific, “inner” surface refers to the regions of difficult accessibility, and “outer” capacity mainly comes from the surface exposed directly to ions. The calculation is based on Equations (7) and (8) [44].

(7) $$\begin{array}{c} q^{*}=q_{\text{s},\text{out}}+A_{1}v^{-1/2}, \end{array}$$

(8) $$\begin{array}{c} q^{*-1}={q_{\text{s}}}^{-1}+A_{2}v^{1/2}. \end{array}$$

where *q*^∗^ is the voltammetric charge, *q*_s_ is the surface-controlled capacity, and *q*_s,out_ is the “outer” capacity. *q*_s,out_ is calculated to be 22 F·g^−1^·cm^−2^, constituting 50% of *q*_s_, which is comparable to modified carbon electrodes [59] and notably higher than CuAl-LDH@rGO [53]. This indicates that hollow Co(OH)_2_ electrode provides rapid, capacitor-like charge transfer and ion removal (Figures 6(c) and 6(d)).

**Figure 7:**
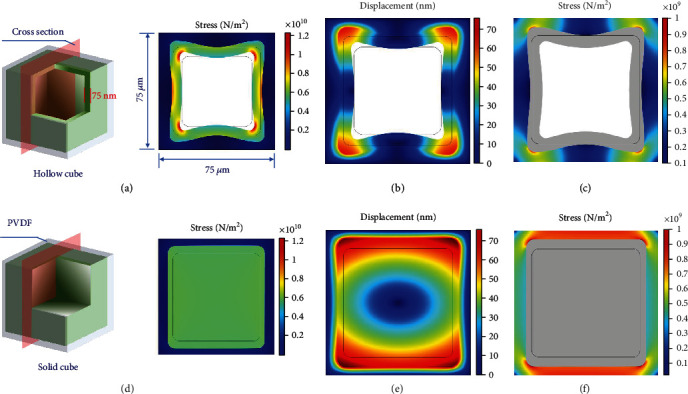
Finite element simulation results of stress and deformation displacement distribution in a hollow cube (a, b) and solid cube (d, e) at a fixed volume expansion percentage. Finite element simulation results of stress distribution in surrounding adhesive PVDF of a hollow cube (c) and solid cube (f).

To further elaborate the cushion effect of hollow configuration during ion intercalation, finite element simulation was carried out to investigate and compare the stress distribution in hollow and solid cubes after volume expansion; detailed simulation information is given in Supporting Information, Text S1). Finite element simulation is applied to analyze the ion concentration distribution during desalination and to demonstrate that a hollow structure is favorable for ion transport [60, 61]. Based on in situ dilatometry, the expansion proportion is fixed at 40% for both structures. For a solid cube, the stress inside the materials is homogeneous at about 0.6 × 10^10^ N/m^2^ after electrochemical volume expansion (Figure 7(d)), while the stress in a hollow cube is heterogeneous. Edges (especially top and bottom edges) of the two-dimensional hollow cross section generally display more minor stress at around 0.4 × 10^10^ N/m^2^. More significant stress (1 × 10^10^ N/m^2^) occurs at the corner (Figure 7(a)) due to stress from adjacent edges and induced shear stress during Na^+^ intercalation. During expansion, the physical deformation of materials is expressed as displacement, that is, bias relative to the initial position. The displacement in a hollow cube is generally smaller, and the maximum value is approximately 60 nm (Figure 7(b)). Contrarily, half of the solid cube experiences remarkable displacement with the maximum deformation of 70 nm (Figure 7(e)). Therefore, the hollow structure is more stable as it undergoes less distortion and stress when charging, except for higher stress at the corner.

The stress distribution of wrapped binder PVDF is studied to reflect the complete working condition of the electrode. The PVDF around the hollow cube exhibits stress at a scale of 0.2 × 10^9^ N/m^2^ (Figure 7(c)), which is generally smaller than the solid cube counterpart (0.9 × 10^9^ N/m^2^ at top and bottom edges; Figure 7(f)). The hollow structure exerts minor stress to surrounding binder material, conducive to keep the whole electrode intact during electrochemical ion intercalation by relieving the accompanying stress.

## 4. Conclusions

In summary, a hollow pseudocapacitive cobalt hydroxide was prepared via facile synthesis conditions. The hollow Co(OH)_2_ cube with vertical nanosheets anchored on the surface has a large ion accessible surface area based on electrochemical analyses, ensuring fast ion transport. Meanwhile, this configuration can relieve the pressure during ion intercalation to enhance the cyclability, proved by finite element simulation. Consequently, h-Co(OH)_2_ electrode displays a high desalination rate of 3.3 mg (NaCl)·g^−1^ (h-Co(OH)_2_)·min^−1^ at 100 mA·g^−1^ and sustains over 90% of the initial capacity after 100 desalination cycles in HCDI cell. Moreover, this hollow electrode holds high capacity intrinsically by avoiding “dead mass,” with the maximum value of 117 ± 6 mg (NaCl)·g^−1^ (h-Co(OH)_2_). The outstanding desalination performance of the hollow structure in the microscale navigates a new direction to the development of Faradaic electrodes for CDI.

## Data Availability

The .txt or .jpeg data used to support the findings of this study are available from the corresponding author upon request.

## Supplementary Materials

Supplementary Materials Fig. S1: transmission electron micrographs of Cu_2_O template etching and Co(OH)_2_ crystal growth process. Fig. S2: water contact angle of the cobalt hydroxide electrode before (A) and after (B) desalination. Fig. S3: cyclic voltammogram of the cobalt hydroxide electrode at a rate of 1 mV·s-1. Fig. S4: (A) cyclic voltammograms of the cobalt hydroxide electrode at a rate of 10 mV·s-1 using a two electrode setup with an oversized AC electrode as counter electrode. (B) Cyclic voltammograms with different cutoff voltages. (C) Charging capacity of Co(OH)_2_ electrode in two electrode system with changing specific current. Fig. S5: desalination capacity (A), cell voltage profiles, (B) and current profiles (C) of the HCDI cell at different specific current. Fig. S6: energy consumption (A) and charge efficiency (B) at different rates. Fig. S7: (A) desalination capacity at 100 mM initial NaCl concentration. (B) Desalination capacity of HCDI cell at different cutoff voltages. Fig. S8: voltage profiles of HCDI cell at different cutoff voltages. Fig. S9: Kim-Yoon plot of h-Co(OH)_2_ electrode in HCDI cell. Fig. S10: electrochemical characterizations for h-Co(OH)_2_ electrodes after desalination. Fig. S11: CV curves and capacitance of h-Co(OH)2 electrode at different voltage windows. Fig. S12: de-chlorination performance. Fig. S13: (A) desalination capacity of symmetric CDI cell for 100 cycles. (B) Theoretical de-chlorination capacity of asymmetric and symmetric CDI cells for 100 cycles. Fig. S14: cell voltage (A) and current profiles (B) of asymmetric cell for 100 cycles. Fig. S15: cell voltage (A) and current profiles (B) of symmetric CDI cell for 100 cycles. Fig. S16: schematic drawing of the in-situ electrochemical dilatometry cell. Fig. S17: (A) the original data of relative strain during electro-dilatometry in CV mode. (B) The relative strain change in the test after 72 h stabilization. Table S1: comparison of desalination rate of various advanced electrode materials. Text S1: finite element simulation.
