# Corrigendum to “Three-Dimensional Cobalt Hydroxide Hollow Cube/Vertical Nanosheets with High Desalination Capacity and Long-Term Performance Stability in Capacitive Deionization”

**DOI:** 10.34133/2021/9809407

**Published:** 2021-12-01

**Authors:** Yuecheng Xiong, Fei Yu, Stefanie Arnold, Lei Wang, Volker Presser, Yifan Ren, Jie Ma

**Affiliations:** ^1^State Key Laboratory of Pollution Control and Resource Reuse, College of Environmental Science and Engineering, Tongji University, Shanghai 200092, China; ^2^Research Center for Environmental Functional Materials, College of Environmental Science and Engineering, Tongji University, 1239 Siping Road, Shanghai 200092, China; ^3^College of Marine Ecology and Environment, Shanghai Ocean University, Shanghai 201306, China; ^4^INM-Leibniz Institute for New Materials, 66123 Saarbrücken, Germany; ^5^Department of Materials Science and Engineering, Saarland University, 66123 Saarbrücken, Germany; ^6^Saarene-Saarland Center for Energy Materials and Sustainability, 66123 Saarbrücken, Germany; ^7^Shanghai Institute of Pollution Control and Ecological Security, Shanghai 200092, China

In the article titled “Three-Dimensional Cobalt Hydroxide Hollow Cube/Vertical Nanosheets with High Desalination Capacity and Long-Term Performance Stability in Capacitive Deionization” [1], there was an error in the title. It previously read “Three-Dimensional Cobalt Hydroxide Hollow Cube/Vertical Nanosheets with High Desalination Capacity and Long-Term Performance Stability”, which was incorrect. The title in the original version has now been updated.

## References

[1] Xiong Y., Yu F., Arnold S. (2021). Three-Dimensional Cobalt Hydroxide Hollow Cube/Vertical Nanosheets with High Desalination Capacity and Long-Term Performance Stability in Capacitive Deionization. *Research*.

