# Supplementary material for: Three-Dimensional Cobalt Hydroxide Hollow Cube/Vertical Nanosheets with High Desalination Capacity and Long-Term Performance Stability in Capacitive Deionization
Source: Research (Wash D C). 2021 Oct 26;2021:9754145. doi: 10.34133/2021/9754145 (PMC8566195; doi:10.34133/2021/9754145)
Supplement: Supplementary Materials — Fig. S1: transmission electron micrographs of Cu2O template etching and Co(OH)2 crystal growth process. Fig. S2: water contact angle of the cobalt hydroxide electrode before (A) and after (B) desalination. Fig. S3: cyclic voltammogram of the cobalt hydroxide electrode at a rate of 1 mV·s-1. Fig. S4: (A) cyclic voltammograms of the cobalt hydroxide electrode at a rate of 10 mV·s-1 using a two electrode setup with an oversized AC electrode as counter electrode. (B) Cyclic voltammograms with different cutoff voltages. (C) Charging capacity of Co(OH)2 electrode in two electrode system with changing specific current. Fig. S5: desalination capacity (A), cell voltage profiles, (B) and current profiles (C) of the HCDI cell at different specific current. Fig. S6: energy consumption (A) and charge efficiency (B) at different rates. Fig. S7: (A) desalination capacity at 100 mM initial NaCl concentration. (B) Desalination capacity of HCDI cell at different cutoff voltages. Fig. S8: voltage profiles of HCDI cell at different cutoff voltages. Fig. S9: Kim-Yoon plot of h-Co(OH)2 electrode in HCDI cell. Fig. S10: electrochemical characterizations for h-Co(OH)2 electrodes after desalination. Fig. S11: CV curves and capacitance of h-Co(OH)2 electrode at different voltage windows. Fig. S12: de-chlorination performance. Fig. S13: (A) desalination capacity of symmetric CDI cell for 100 cycles. (B) Theoretical de-chlorination capacity of asymmetric and symmetric CDI cells for 100 cycles. Fig. S14: cell voltage (A) and current profiles (B) of asymmetric cell for 100 cycles. Fig. S15: cell voltage (A) and current profiles (B) of symmetric CDI cell for 100 cycles. Fig. S16: schematic drawing of the in-situ electrochemical dilatometry cell. Fig. S17: (A) the original data of relative strain during electro-dilatometry in CV mode. (B) The relative strain change in the test after 72 h stabilization. Table S1: comparison of desalination rate of various advanced electrode materials. Text S1: fini [file 9754145.f1.docx]

**Supporting Information**

**Three-dimensional hollow Co(OH)_2_ cube/vertical nanosheets
with** **high desalination capacity and long-term performance stability**

Yuecheng Xiong^2,6§^, Fei Yu^1§^, Stefanie Arnold^3,4^, Lei Wang^3,4^, Volker Presser^3,4,5^, Yifan Ren^2,6^, Jie Ma^2,6,7*^

1 College of Marine Ecology and Environment, Shanghai Ocean University, Shanghai 201306, P. R. China

2 Research Center for Environmental Functional Materials, College of Environmental Science and Engineering, Tongji University, 1239 Siping Road, Shanghai 200092, P.R. China

3 INM-Leibniz Institute for New Materials, 66123 Saarbrücken, Germany

4 Department of Materials Science and Engineering, Saarland University, 66123 Saarbrücken, Germany

5 Saarene-Saarland Center for Energy Materials and Sustainability, 66123 Saarbrücken, Germany.

6 State Key Laboratory of Pollution Control and Resource Reuse, College of Environmental Science and Engineering, Tongji University, Shanghai, 200092, P. R. China.

7 Shanghai Institute of Pollution Control and Ecological Security, Shanghai, 200092, P.R. China

§ These authors contributed equally to this work

* Corresponding author’s email: [jma@tongji.edu.cn](mailto:jma@tongji.edu.cn)

**Contents**

**Fig. S1.** Transmission electron micrographs of Cu_2_O template etching and Co(OH)_2_ crystal growth process.

**Fig. S2.** Water contact angle of the cobalt hydroxide electrode before (A) and after (B) desalination.

**Fig. S3.** Cyclic voltammogram of the cobalt hydroxide electrode at a rate of 1 mV·s^-1^.

**Fig. S4.** (A) Cyclic voltammograms of the cobalt hydroxide electrode at a rate of 10 mV·s^-1^ using a two electrode setup with an oversized AC electrode as counter electrode. (B) Cyclic voltammograms with different cutoff voltages. (C) Charging capacity of Co(OH)_2_ electrode in two electrode system with changing specific current.

**Fig. S5.** Desalination capacity (A), cell voltage profiles, (B) and current profiles (C) of the HCDI cell at different specific current.

**Fig. S6.** Energy consumption (A) and charge efficiency (B) at different rates.

**Fig. S7.** (A) Desalination capacity at 100 mM initial NaCl concentration. (B) Desalination capacity of HCDI cell at different cutoff voltages.

**Fig. S8.** Voltage profiles of HCDI cell at different cutoff voltages.

**Fig. S9**. Kim-Yoon plot of h-Co(OH)_2_ electrode in HCDI cell.

**Fig. S10.** Electrochemical characterizations for h-Co(OH)_2_ electrodes after desalination.

**Fig. S11.** CV curves and capacitance of h-Co(OH)_2_ electrode at different voltage windows.

**Fig. S12.** De-chlorination performance.

**Fig. S13.** (A) Desalination capacity of symmetric CDI cell for 100 cycles. (B) Theoretical de-chlorination capacity of asymmetric and symmetric CDI cells for 100 cycles.

**Fig. S14.** Cell voltage (A) and current profiles (B) of asymmetric cell for 100 cycles.

**Fig. S15.** Cell voltage (A) and current profiles (B) of symmetric CDI cell for 100 cycles.

**Fig. S16.** Schematic drawing of the in-situ electrochemical dilatometry cell.

**Fig. S17.** (A) The original data of relative strain during electro-dilatometry in CV mode. (B) The relative strain change in the test after 72 h stabilization.

**Table S1** Comparison of desalination rate of various advanced electrode materials.

**Text S1** Finite element simulation.


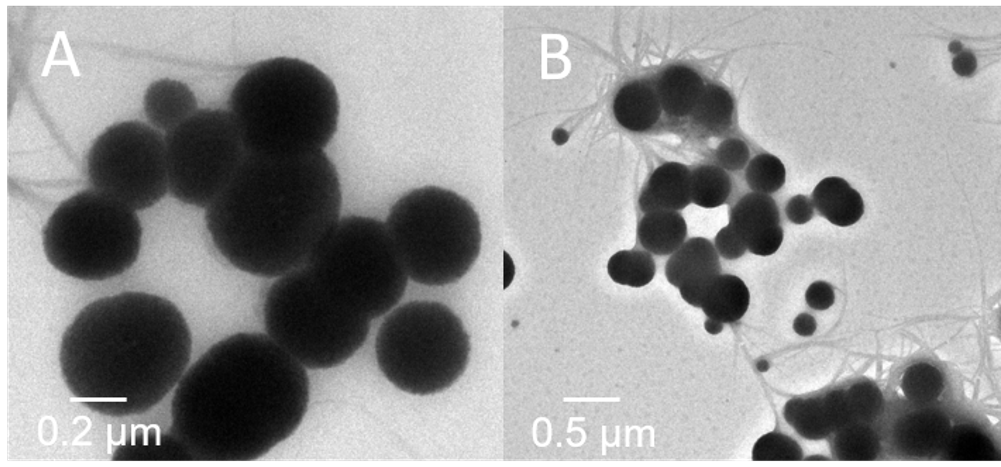


**Fig. S1.** Transmission electron micrographs of Cu_2_O template etching and cobalt hydroxide crystal growth process.

**
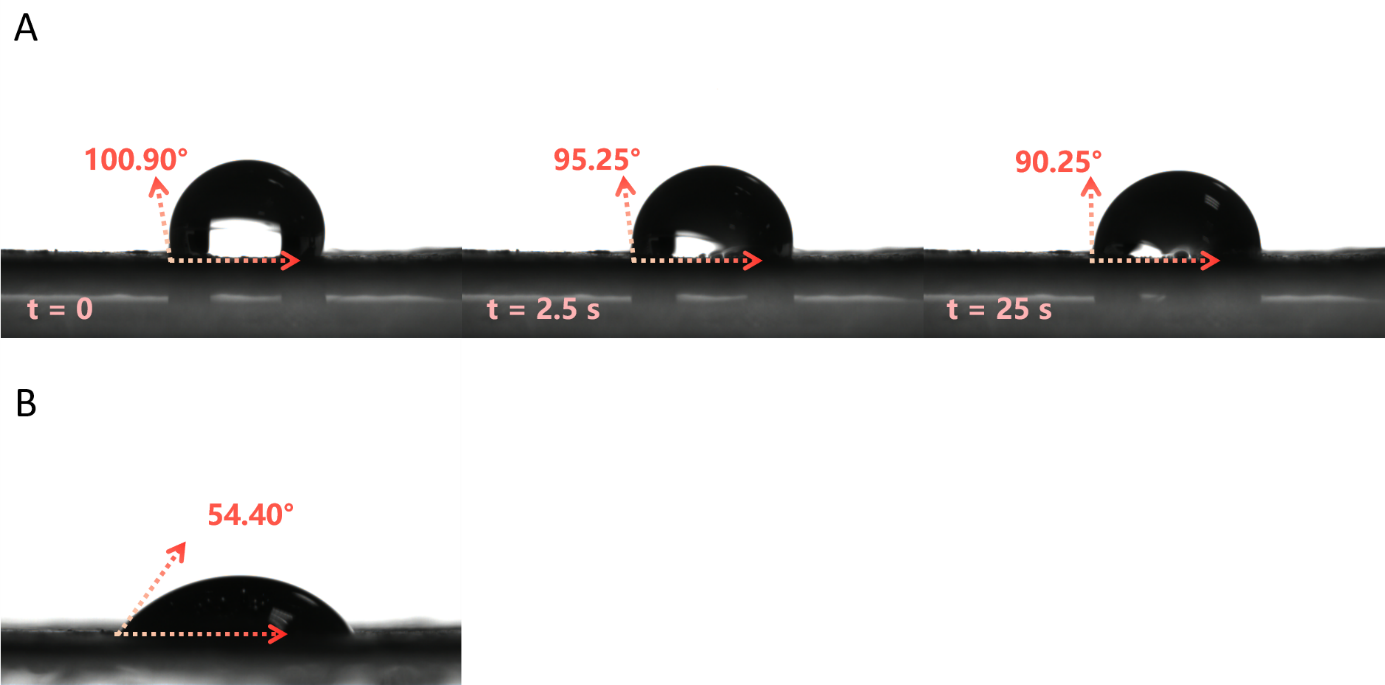
**

**Fig. S2.** Water contact angle of the cobalt hydroxide _2_ electrode before (A) and after (B) desalination.





**Fig. S3.** Cyclic voltammogram of the cobalt hydroxide electrode at a rate of 1 mV·s^-1^.

**
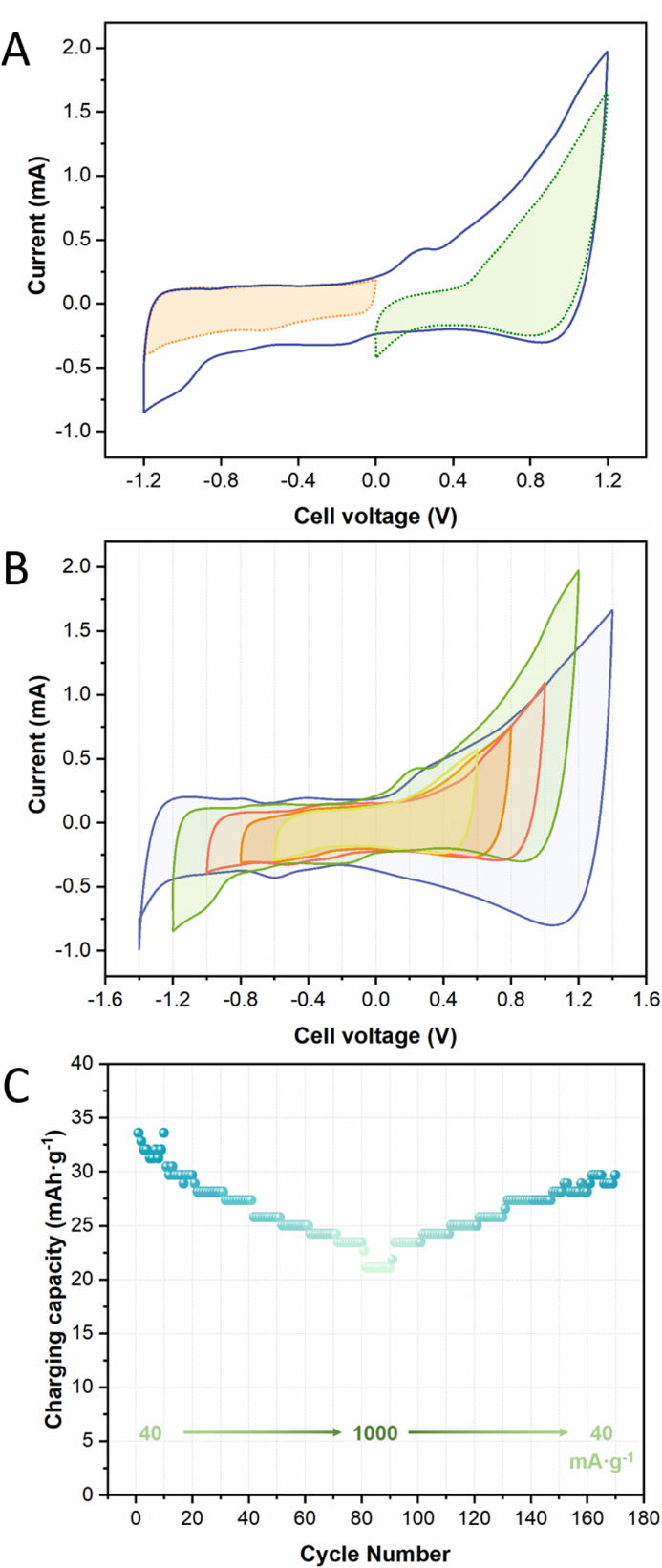
**

**Fig. S4.** (A) Cyclic voltammograms of the cobalt hydroxide electrode at a rate of 10 mV·s^-1^ using a two-electrode setup with an oversized AC electrode as counter electrode. (B) Cyclic voltammograms with different cutoff voltages. (C) Charging capacity of the cobalt hydroxide electrode in two electrode system with changing specific current.

**
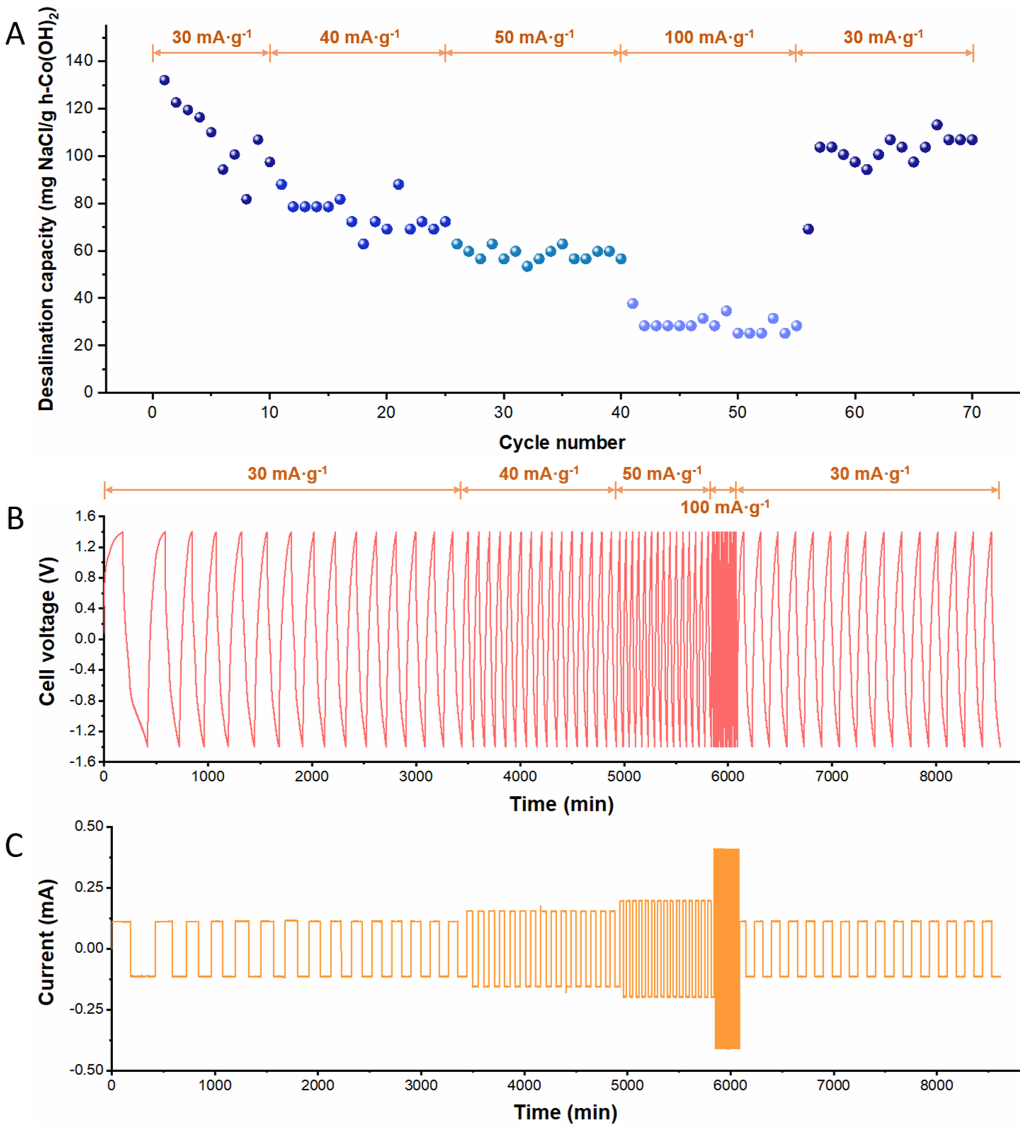
**

**Fig. S5.** Desalination capacity (A), cell voltage profiles, (B) and current profiles (C) of the HCDI cell at different specific current.


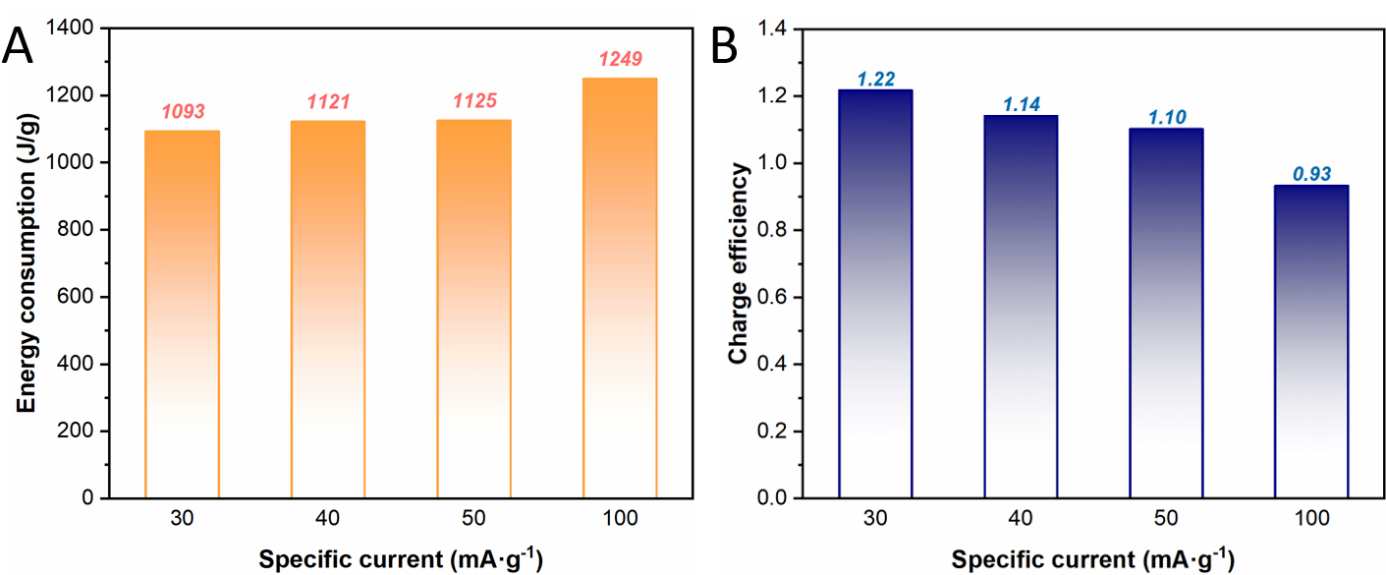


**Fig. S6.** Energy consumption (A) and charge efficiency (B) at different rates.


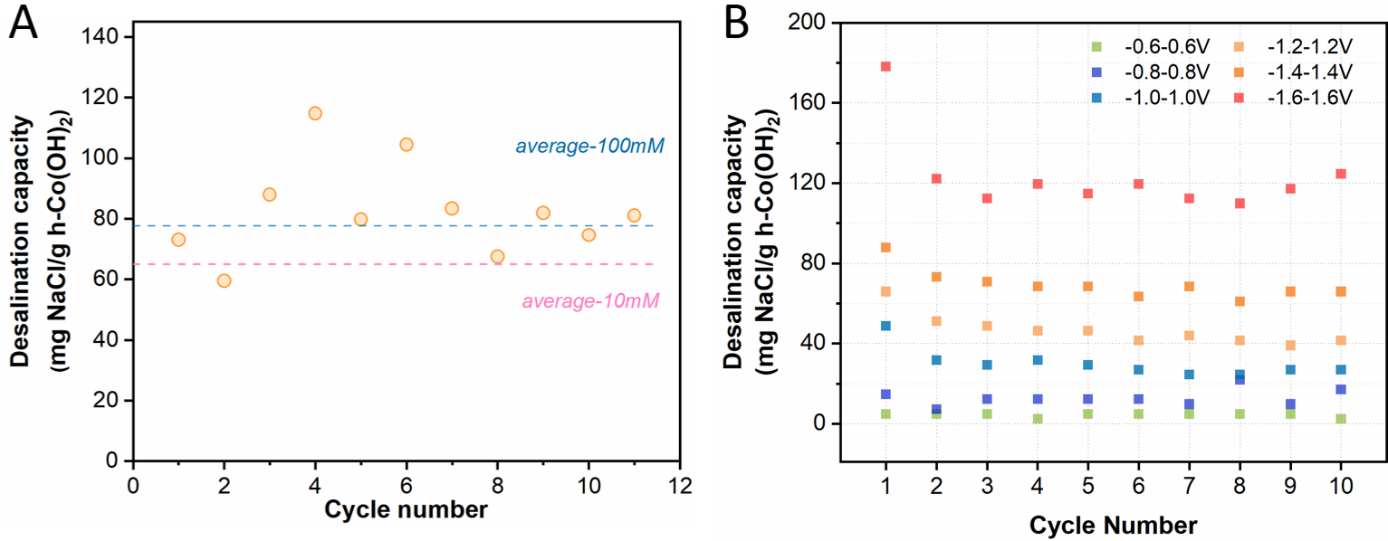


**Fig. S7.** (A) Desalination capacity at 100 mM initial NaCl concentration (specific current: 30 mA·g^-1^; cutoff voltage: ±1.4 V). (B) Desalination capacity of HCDI cell at different cutoff voltages (specific current: 30 mA·g^-1^; initial NaCl concentration: 10 mM).

**

**

**Fig. S8.** Voltage profiles of the HCDI cell at different cutoff voltages.


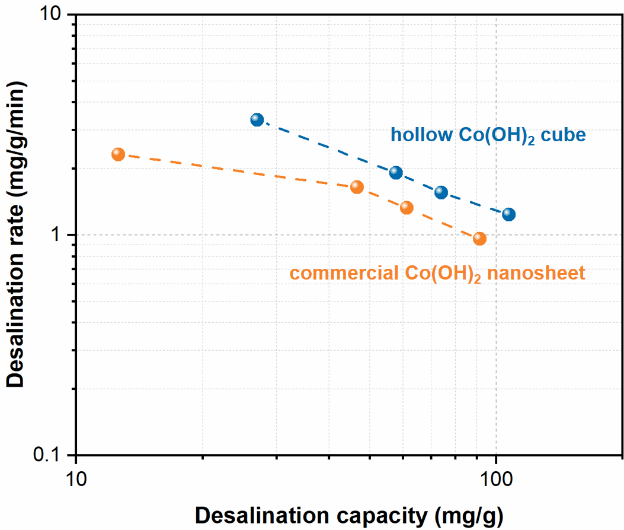


**Fig. S9**. Kim-Yoon plot of h-Co(OH)_2_ and commercial Co(OH)_2_ electrode in an HCDI cell.


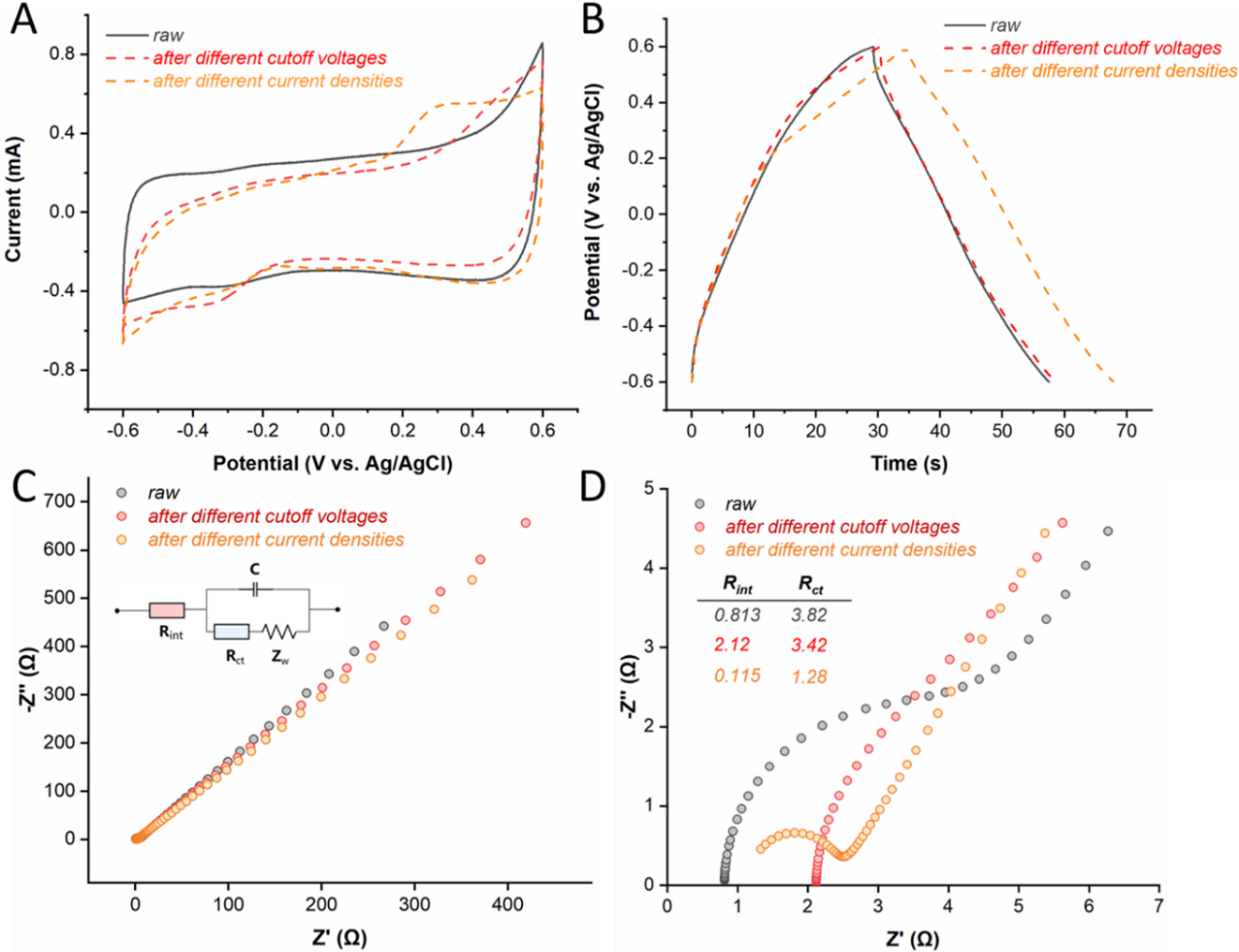


**Fig. S10.** Cyclic voltammograms (A), galvanostatic charge/discharge profiles (B), Nyquist plots (C) and magnified Nyquist plots at high-frequency area (D) for cobalt hydroxide electrodes before and after desalination at different cutoff voltages and values for specific current.

***De-chlorination performance.***

The capacitance during positive and negative polarization increases as the potential difference is increased during cyclic voltammetry (**Fig. S11**). A similar capacitance during opposite polarization indicates the ability of h-Co(OH)_2_ electrode to serve as an electrode for the removal of either anions or cations.^1^ The Cl^-^ insertion has been proved in cobalt layered hydroxide, with the formula presented below.^2^

Co(OH)_2_ + Cl^-^ → Co(OH)_2_Cl + e^-^

In our experiments, we further paired two h-Co(OH)_2_ electrodes to investigate its de-chlorination potential in capacitive deionization. The average desalination capacity of the symmetric cell improves to 80 mg·g^-1^, while the counterpart for HCDI is 70 mg·g^-1^ (**Fig. S12**). The average de-chlorination capacity based on anode mass increases slightly from 36 mg·g^-1^ to 39 mg·g^-1^. This performance is higher than that found for conventional activated carbon, while the symmetric cell displays a higher instability with wider lower-upper limit and more outliers (**Fig. S13-S15**).





**Fig. S11.** Cyclic voltammograms and capacitance of h-Co(OH)_2_ electrode at different voltage windows.


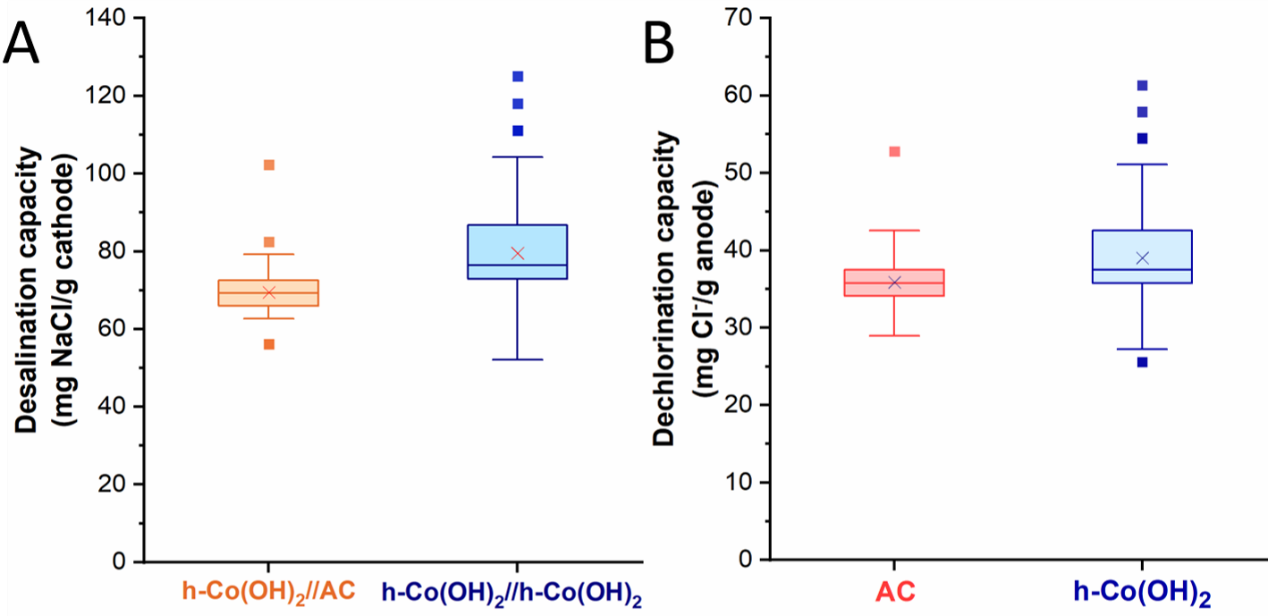


**Fig. S12.** De-chlorination performance. (A) Desalination capacity of asymmetric (h-Co(OH)_2_//AC) and symmetric (h-Co(OH)_2_//h-Co(OH)_2_) CDI cells. (B) De-chlorination capacity of the asymmetric and symmetric CDI cells.


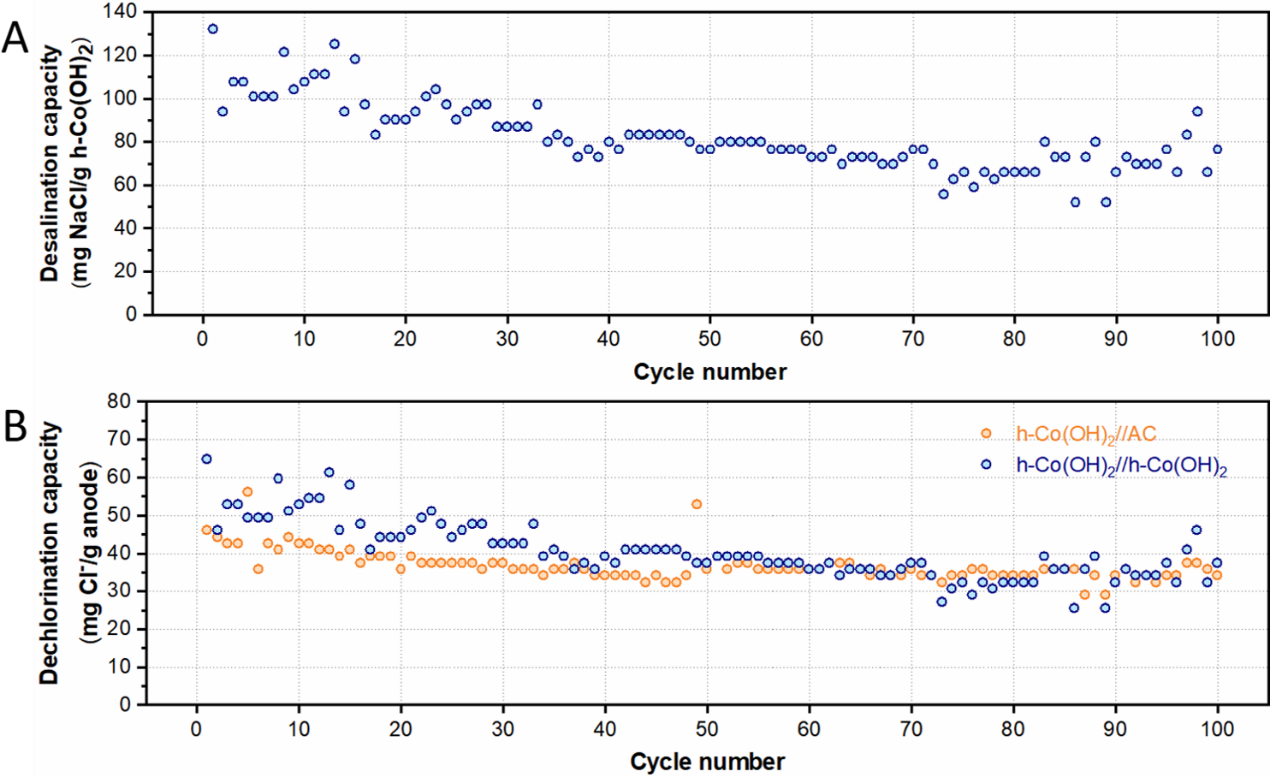


**Fig. S13.** (A) Desalination capacity of symmetric CDI cell for 100 cycles. (B) Theoretical de-chlorination capacity of asymmetric and symmetric CDI cells for 100 cycles.

**
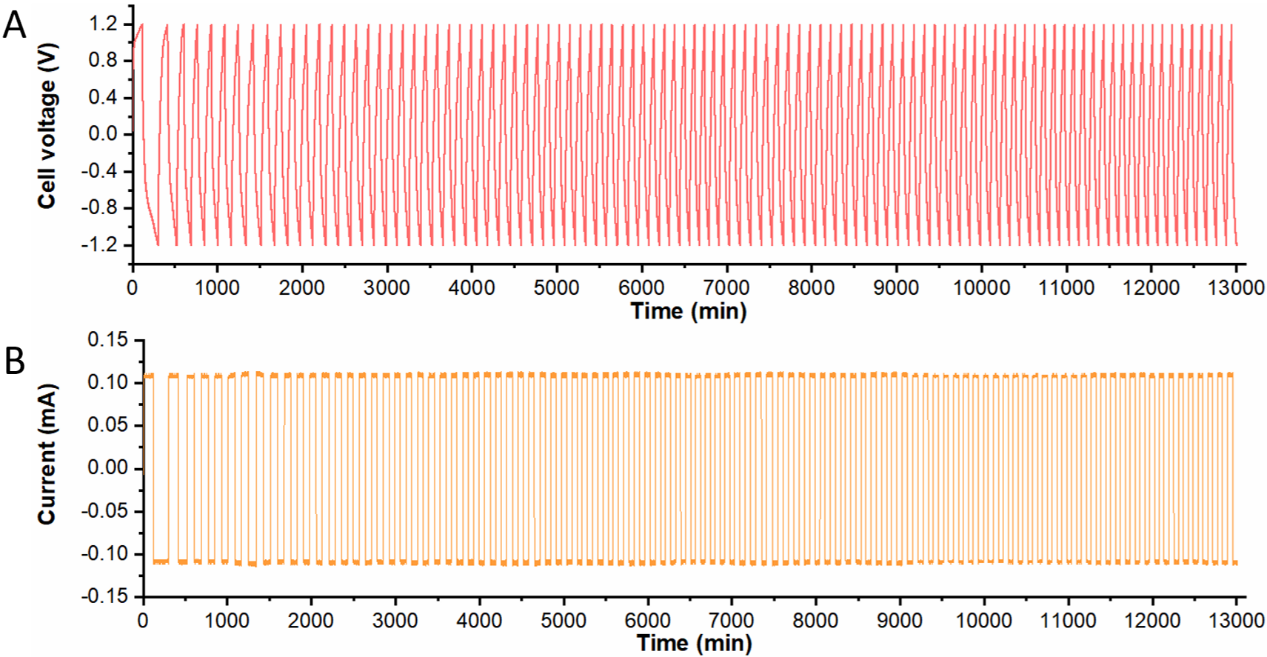
**

**Fig. S14.** Cell voltage (A) and current profiles (B) of asymmetric cell for 100 cycles.


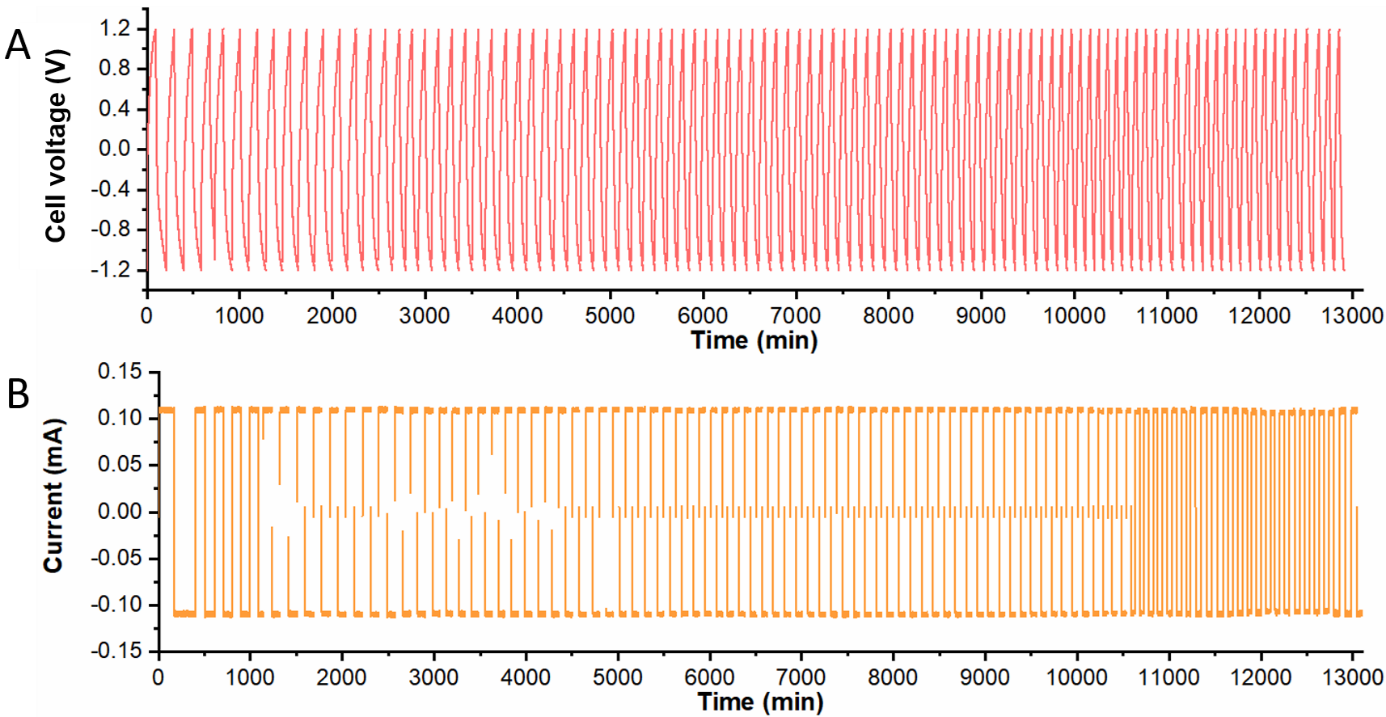


**Fig. S15.** Cell voltage (A) and current profiles (B) of symmetric CDI cell for 100 cycles.


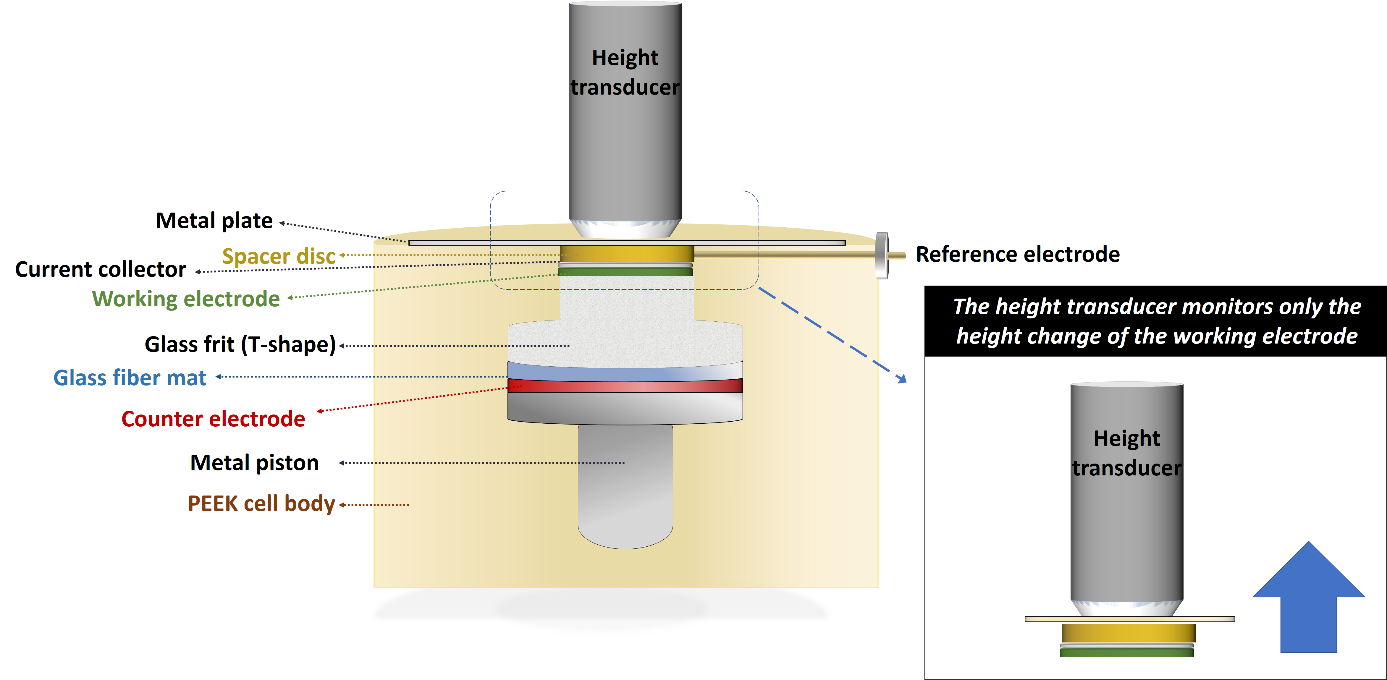


**Fig. S16.** Schematic drawing of the in-situ electrochemical dilatometry cell.


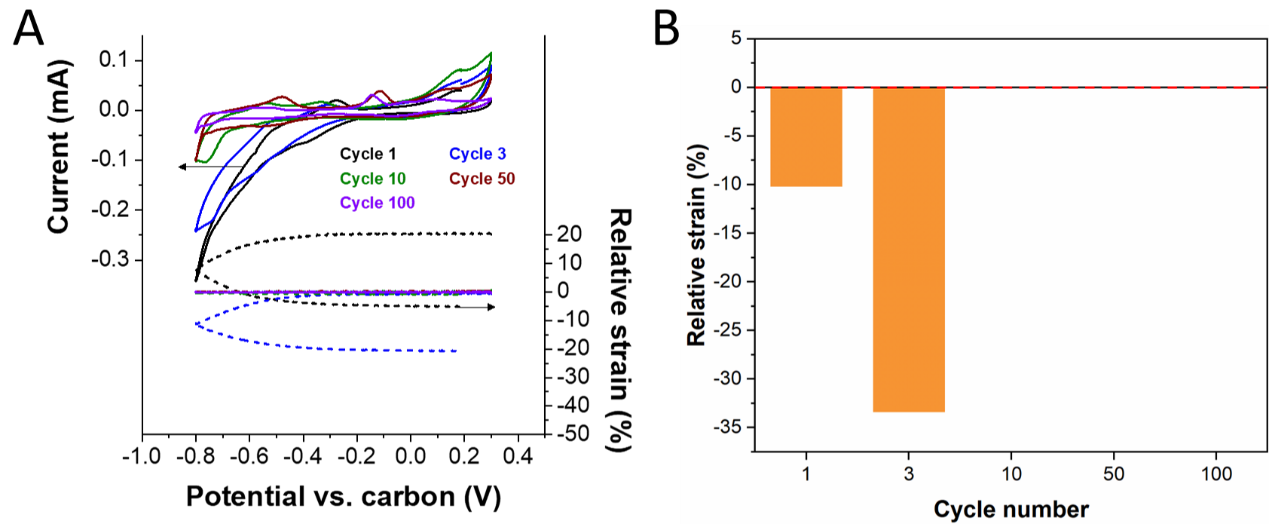


**Fig. S17.** (A) The original data of relative strain during electro-dilatometry during cyclic voltammetry. (B) The relative strain change in the test after 72 h stabilization.

**Table S1** Comparison of desalination rate of various advanced electrode materials

| **Materials** | **Testing conditions** | **Desalination rate**  **(mg·g^-1^·s^-1^)** | **Ref.** |
| --- | --- | --- | --- |
| PB/PANI | 500 ppm, 100 mA·g^-1^ | 0.03 | ^3^ |
| h-Co(OH)_2_ | 10 mM, 100 mA·g^-1^ | 0.055 | This work |
| N-Ti_3_C_2_T*_x_* | 5000 ppm, 1.2 V | 0.062 | ^4^ |
| Na_3_V_2_(PO_4_)_3_@C | 10 mM, 1 V | 0.04 | ^5^ |
| K_0.03_Cu[Fe(CN)_6_]_0.65_·0.43H_2_O | 500 ppm, 1.2 V | 0.24 | ^6^ |
| MnO_2_ | 15 mM, 1.2 V | 0.112 | ^7^ |
| ZIF-67/PPy | 10 mM, 1.2 V | 0.019 | ^8^ |

**N.B.** The pink and yellow backgrounds represent the constant current and constant voltage mode, respectively. 10 mM NaCl equals to ca. 585 ppm NaCl. The estimation of the desalination rate in ref [4] and [8] takes the equilibrium time of 700 sec and 600 sec, respectively. PB, PANI, and PPy refer to Prussian blue, polyaniline and polypyrrole, respectively.

**Text S1 Finite element simulation**

The finite element simulation method is based on COMSOL software, combining the structural mechanics and heat transfer modules. In the heat transfer module, the thermal expansion coefficient is applied as an approximate substitution of cobalt hydroxide material expansion or contraction induced by sodium ion intercalation/deintercalation. Through the simultaneous setting of thermal expansion coefficient and the temperature K, the volume change proportion is set as 40%.

First, a hollow cube and solid cube model are constructed, with a dimension of 75 ×75 μm^2^, and the wall thickness of the hollow cube is 75 nm. The density of Co(OH)_2_ is set as 3.6 g·cm^-3^, and Co(OH)_2_ is an anisotropic material, which is expressed as the following elasticity matrix.


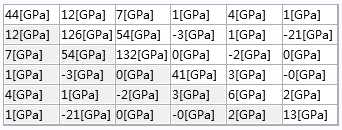


For PVDF, we assume a density of 1.77 g·cm^-3^, the Young’s modulus is 2000 MPa, Poisson’s ratio is 0.3, and an isotropic behavior. The initial displacement and velocity is set as 0, and the boundary conditions are fixed, that is, the displacement is 0.

Elastic stress follows the Hook’s law:

$$\varepsilon_{ij}^{e}=\frac{1}{E}\left[ \left( 1+v \right)\sigma_{ij}-v\sigma_{kk}\delta_{ij} \right]$$

where ε_ij_ is the stress fractional variable, σ_ij_ is the strain fractional variable, E is the Young’s modulus, *v* is the Poisson’s ratio, δ is the second-order unit tensor.

**References**

1. Torkamanzadeh, M.; Wang, L.; Zhang, Y.; Budak, O.; Srimuk, P.; Presser, V., MXene/Activated-Carbon Hybrid Capacitive Deionization for Permselective Ion Removal at Low and High Salinity. *ACS Appl. Mater. Interfaces* **2020,** *12*, (23), 26013-26025.

2. Young, M. J.; Kiryutina, T.; Bedford, N. M.; Woehl, T. J.; Segre, C. U., Discovery of Anion Insertion Electrochemistry in Layered Hydroxide Nanomaterials. *Sci Rep* **2019,** *9*, (1), 2462.

3. Shi, W.; Liu, X.; Deng, T.; Huang, S.; Ding, M.; Miao, X.; Zhu, C.; Zhu, Y.; Liu, W.; Wu, F.; Gao, C.; Yang, S. W.; Yang, H. Y.; Shen, J.; Cao, X., Enabling Superior Sodium Capture for Efficient Water Desalination by a Tubular Polyaniline Decorated with Prussian Blue Nanocrystals. *Adv. Mater.* **2020,** *32*, (33), e1907404.

4. Amiri, A.; Chen, Y.; Bee Teng, C.; Naraghi, M., Porous nitrogen-doped MXene-based electrodes for capacitive deionization. *Energy Storage Mater.* **2020,** *25*, 731-739.

5. Cao, J.; Wang, Y.; Wang, L.; Yu, F.; Ma, J., Na3V2(PO4)3@C as Faradaic Electrodes in Capacitive Deionization for High-Performance Desalination. *Nano Lett* **2019,** *19*, (2), 823-828.

6. Choi, S.; Chang, B.; Kim, S.; Lee, J.; Yoon, J.; Choi, J. W., Battery Electrode Materials with Omnivalent Cation Storage for Fast and Charge-Efficient Ion Removal of Asymmetric Capacitive Deionization. *Adv. Funct. Mater.* **2018,** *28*, (35), 1802665.

7. Byles, B. W.; Cullen, D. A.; More, K. L.; Pomerantseva, E., Tunnel structured manganese oxide nanowires as redox active electrodes for hybrid capacitive deionization. *Nano Energy* **2018,** *44*, 476-488.

8. Wang, Z.; Xu, X.; Kim, J.; Malgras, V.; Mo, R.; Li, C.; Lin, Y.; Tan, H.; Tang, J.; Pan, L.; Bando, Y.; Yang, T.; Yamauchi, Y., Nanoarchitectured metal–organic framework/polypyrrole hybrids for brackish water desalination using capacitive deionization. *Mater. Horiz.* **2019**.
